# Simulation of spreading depolarization trajectories in cerebral cortex: Correlation of velocity and susceptibility in patients with aneurysmal subarachnoid hemorrhage

**DOI:** 10.1016/j.nicl.2017.09.005

**Published:** 2017-09-06

**Authors:** Denny Milakara, Cristian Grozea, Markus Dahlem, Sebastian Major, Maren K.L. Winkler, Janos Lückl, Michael Scheel, Vasilis Kola, Karl Schoknecht, Svetlana Lublinsky, Alon Friedman, Peter Martus, Jed A. Hartings, Johannes Woitzik, Jens P. Dreier

**Affiliations:** a Center for Stroke Research, Charité – Universitätsmedizin Berlin, corporate member of Freie Universität Berlin, Humboldt-Universität zu Berlin, and Berlin Institute of Health, Berlin, Germany; b VISCOM – Visual Computing at Fraunhofer Institute for Open Communication Systems FOKUS, Berlin, Germany; c Department of Physics, Humboldt-University Berlin, Berlin, Germany; d Department of Neurology, Charité – Universitätsmedizin Berlin, corporate member of Freie Universität Berlin, Humboldt-Universität zu Berlin, and Berlin Institute of Health, Berlin, Germany; e Department of Experimental Neurology, Charité – Universitätsmedizin Berlin, corporate member of Freie Universität Berlin, Humboldt-Universität zu Berlin, and Berlin Institute of Health, Berlin, Germany; f Department of Neuroradiology, Charité – Universitätsmedizin Berlin, corporate member of Freie Universität Berlin, Humboldt-Universität zu Berlin, and Berlin Institute of Health, Berlin, Germany; g Department of Physiology and Neurobiology, Faculty of Health Sciences and Zlotowski Center for Neuroscience, Ben-Gurion University of the Negev, Beer-Sheva, Israel; h Department of Medical Neuroscience, Faculty of Medicine, Dalhousie University, Halifax, Canada; i Institute for Clinical Epidemiology and Applied Biometry, University of Tübingen, Tübingen, Germany; j Department of Neurosurgery, University of Cincinnati College of Medicine, Cincinnati, OH, USA; k Department of Neurosurgery, Charité – Universitätsmedizin Berlin, corporate member of Freie Universität Berlin, Humboldt-Universität zu Berlin, and Berlin Institute of Health, Berlin, Germany

**Keywords:** AC, alternating current, ADC, apparent diffusion coefficient, aSAH, aneurysmal subarachnoid hemorrhage, COSBID, Co-Operative Studies on Brain Injury Depolarizations, CT, computed tomography, DC, direct current, DWI, diffusion-weighted imaging, E, electrode, ECoG, electrocorticography, FLAIR, fluid-attenuated inversion recovery, HU, Hounsfield units, ICH, intracerebral hemorrhage, IOS, intrinsic optical signal, MCA, middle cerebral artery, MHS, malignant hemispheric stroke, MPRAGE, magnetization prepared rapid gradient echo, MRI, magnetic resonance imaging, NO, nitric oxide, PTDDD, peak total SD-induced depression duration of a recording day, R_diff, radius difference, SAH, subarachnoid hemorrhage, SD, spreading depolarization, SPC, slow potential change, TBI, traumatic brain injury, TOAD, time-of-SD-arrival-difference, V_diff, velocity difference, WFNS, World Federation of Neurosurgical Societies, 3D, three dimensional, Cytotoxic edema, Ischemia, Spreading depression, Stroke, Subarachnoid hemorrhage, Traumatic brain injury

## Abstract

In many cerebral grey matter structures including the neocortex, spreading depolarization (SD) is the principal mechanism of the near-complete breakdown of the transcellular ion gradients with abrupt water influx into neurons. Accordingly, SDs are abundantly recorded in patients with traumatic brain injury, spontaneous intracerebral hemorrhage, aneurysmal subarachnoid hemorrhage (aSAH) and malignant hemispheric stroke using subdural electrode strips. SD is observed as a large slow potential change, spreading in the cortex at velocities between 2 and 9 mm/min. Velocity and SD susceptibility typically correlate positively in various animal models. In patients monitored in neurocritical care, the Co-Operative Studies on Brain Injury Depolarizations (COSBID) recommends several variables to quantify SD occurrence and susceptibility, although accurate measures of SD velocity have not been possible. Therefore, we developed an algorithm to estimate SD velocities based on reconstructing SD trajectories of the wave-front's curvature center from magnetic resonance imaging scans and time-of-SD-arrival-differences between subdural electrode pairs. We then correlated variables indicating SD susceptibility with algorithm-estimated SD velocities in twelve aSAH patients. Highly significant correlations supported the algorithm's validity. The trajectory search failed significantly more often for SDs recorded directly over emerging focal brain lesions suggesting in humans similar to animals that the complexity of SD propagation paths increase in tissue undergoing injury.

## Highlights

• An algorithm has been developed to estimate spreading depolarization (SD) velocities in neurocritical care.
• The algorithm is based on reconstructing SD trajectories of the wave-front's curvature center.
• It utilizes MRI scans and time-of-SD-arrival-differences between subdural electrode pairs.
• Variables indicating SD susceptibility correlated with algorithm-estimated SD velocities.
• The findings establish the opportunity to exploit the SD velocity as part of the multimodal assessment in neurocritical care.

## 1 Introduction

It is now increasingly recognized that spreading depolarization (SD) is the principal mechanism of the mass edema of neurons in many grey matter structures of the brain including the neocortex (Dreier and Reiffurth, 2017). Its hallmark is the abrupt, near-complete, toxic breakdown of the transcellular ion gradients, which creates an osmotic driving force for influx of water (Dreier et al., 2013). Whether or not the SD-induced mass edema, also termed cytotoxic edema, progresses toward cellular injury, is determined by the local tissue conditions and their repercussions on mechanisms involved in the SD process. The term SD continuum describes the changing characteristics of the wave dependent on the local tissue conditions (Dreier and Reiffurth, 2015, Hartings et al., 2017b). The full SD continuum is, for example, observed in a single SD wave when it originates in the center of focal ischemia and subsequently invades first the penumbra and then the surrounding well-nourished tissue against the gradients of oxygen, glucose and perfusion.

SD-induced cytotoxic edema is observed in animals using electron microscopy (Van Harreveld and Khattab, 1967) and the quaternary ammonium salt method (Mazel et al., 2002, Perez-Pinzon et al., 1995, Windmuller et al., 2005) as an abrupt decrease of the extracellular volume fraction and increase in tortuosity, which describes the average path length for diffusion between two points in the extracellular compartment. It is moreover visualized as swelling of the neuronal somata and dendritic beading using two-photon microscopy (Murphy et al., 2008, Obeidat et al., 2000, Rungta et al., 2015, Steffensen et al., 2015, Takano et al., 2007). These structural changes hinder the mobility of water in both the intra- and extracellular compartment during SD no matter whether SD passes through well-perfused or ischemic tissue (Budde and Frank, 2010, Mazel et al., 2002, Perez-Pinzon et al., 1995). On this basis, SD causes abrupt decline in the apparent diffusion coefficient (ADC) of water in diffusion-weighted magnetic resonance imaging (MRI) scans (Cain et al., 2017, de Crespigny et al., 1998, de Crespigny et al., 1999). According to Leão's original notion that SD is the principal response of neurons to a prolonged episode of cerebral ischemia (Leão, 1947, Marshall, 1959), this ADC decline represents the gold standard for diagnosis of acute ischemia in the cortex in clinical neurology (Dreier and Reiffurth, 2015). It corresponds well with this notion that electrophysiological evidence of SDs has been found in practically 100% of patients with malignant hemispheric stroke (MHS) (Dohmen et al., 2008, Woitzik et al., 2013), 70–80% of patients with poor-grade aneurysmal subarachnoid hemorrhage (aSAH) (Dreier et al., 2009, Dreier et al., 2006), 60–70% of patients with intracerebral hemorrhage (ICH) (Fabricius et al., 2006, Helbok et al., 2017) and 50–60% of patients with severe traumatic brain injury (TBI) (Fabricius et al., 2006, Hartings et al., 2011a). Animal experiments suggest that, similar to electrographic seizures, SDs never occur spontaneously in healthy brain (Dreier and Reiffurth, 2017, Hartings et al., 2017b).

**Fig. 1:**
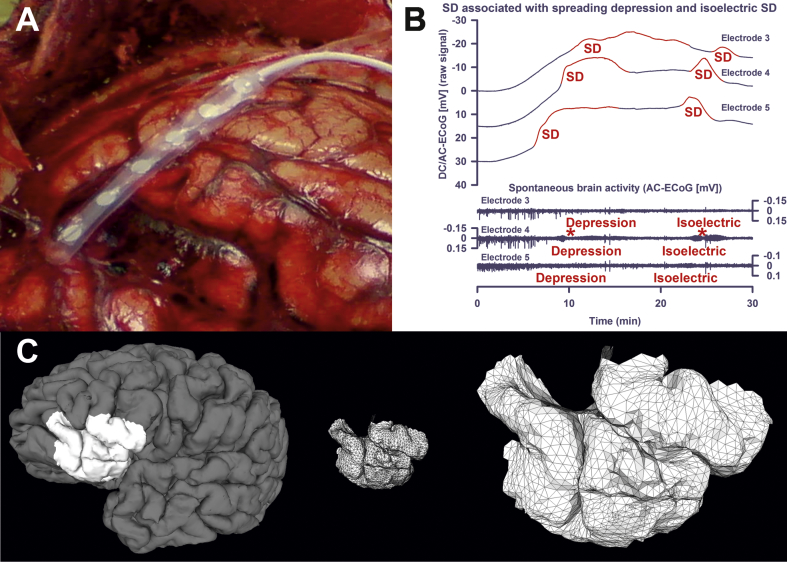
Methodological basis. (A) Wyler electrode strip placed on the cortical surface during craniotomy in a patient with aSAH. Note the subarachnoid blood clot in the lower left region. (B) The first two SDs of a cluster are shown. The first SD starts in electrically active tissue. In electrically active tissue, SD induces spreading depression of activity. Such SDs in electrically active tissue received the epithet “spreading depression”. The second SD starts in tissue that is still electrically inactive after the previous SD. Under this condition, SD is denoted with the adjective “isoelectric” (Dreier et al., 2017). (C) Patch cut out from the brain and discretized in the form of a mesh. The cortical surface is mathematically modeled using a grid of triangles in the three-dimensional space (= tessellation). The corners of the triangles are termed ‘vertices’.

Current gold standard for monitoring SDs in patients with TBI, ICH, aSAH and MHS is electrocorticography (ECoG) with a collinear subdural electrode strip (Dreier et al., 2017) (Fig. 1A). SD is observed as a large, abrupt negative electrical potential shift in the direct current (DC) frequency range of the ECoG below ~ 0.05 Hz, also called slow potential change (SPC) (Fig. 1B) (Dreier et al., 2009, Hartings et al., 2011b, Oliveira-Ferreira et al., 2010). This emanates from differences in depolarization between soma and dendrites (Makarova et al., 2010). The energy-dependent local duration of the DC shift indicates the local duration of mass depolarization and cytotoxic edema and, hence, the risk of injury at the recording site (Back et al., 1994, Dijkhuizen et al., 1999, Hinzman et al., 2015, Nallet et al., 1999, Oliveira-Ferreira et al., 2010). Changes in spontaneous activity are simultaneously recorded in the alternate current (AC) frequency range of the ECoG above ~ 0.5 Hz. In electrically active tissue, SD typically leads to cessation of the spontaneous activity due to sodium channel inactivation, collapse of ion gradients and suppression of synaptic transmission (Kager et al., 2002, Lindquist and Shuttleworth, 2017, Sawant-Pokam et al., 2017). This has been called spreading depression of cortical activity (Fig. 1B) (Leão, 1944).

Clusters of SDs are typically linked to complex global and/or focal neurological deficits (Dreier and Reiffurth, 2015, Dreier et al., 2006). Serial neuroimaging studies suggested that such clusters are associated with ischemic lesion progression, especially when accompanied by prolonged depression of activity (Dreier et al., 2012, Dreier and Reiffurth, 2015, Dreier et al., 2006). Accordingly, SDs in electrically inactive (= isoelectric) tissue, so called isoelectric SDs (Fig. 1B), were associated with poor outcome in patients with TBI (Hartings et al., 2011a) and, in parallel, SDs with prolonged depression periods were linked to worse outcome in patients with aSAH (Dreier et al., 2012, Winkler et al., 2017). Temporal clusters of SDs and persistent depression of spontaneous cortical activity can afford even remote detection of ischemic zones because SDs propagate widely (Dreier et al., 2017, Oliveira-Ferreira et al., 2010, Winkler et al., 2017).

Leão, however, also suggested that a single SD-induced spreading depression of activity in normal and eloquent brain tissue is the pathophysiological correlate of the mostly harmless migraine aura (Leão and Morison, 1945). Case series using imaging of changes in regional cerebral blood flow (rCBF) or its surrogates and magnetoencephalography supported this notion (Bowyer et al., 2001, Hadjikhani et al., 2001, Olesen et al., 1981, Woods et al., 1994). In previous imaging studies of patients undergoing migraine aura, SD velocities of 2.2 (2.1, 2.5 (1st quartile, 3rd quartile)) and 3.5 ± 1.1 mm/min were measured using either the ^133^xenon intracarotideal injection method or functional MRI of changes in the blood oxygen level-dependent (BOLD) signal (Hadjikhani et al., 2001, Lauritzen et al., 1983). In a study of patients with MHS undergoing decompressive hemicraniectomy, SD velocities ranged between 1.7 and 9.2 mm/min using laser speckle imaging of rCBF and imaging of the intrinsic optical signal (IOS) in the operating room (Woitzik et al., 2013). However, in patients undergoing neuromonitoring in critical care, assessment of SD velocities has not been possible until now. While time-of-SD-arrival-differences (TOAD) between adjacent electrodes are known (Strong et al., 2002), the orientation of the SD wavefront relative to the collinear electrode strip and the length of the propagation path along the brain surface between electrodes are unknown. Therefore, in order to estimate SD velocity, we here developed an algorithm for reconstructing SD trajectories of the wave-front's curvature center based on MRI scans and TOADs between different electrode pairs. We then compared the median algorithm-estimated SD velocities of twelve aSAH patients with different variables indicating the tissue's susceptibility to SD such as the median interval between SDs or the peak numbers of SDs, spreading depressions and isoelectric SDs following the recent recommendations of the Co-Operative Studies on Brain Injury Depolarizations (COSBID) (Dreier et al., 2017). Statistically significant correlations supported the validity of the here proposed method.

## 2 Materials and methods

### 2.1 General

Seventy aSAH patients were prospectively enrolled in COSBID between 04/2008 and 09/2012 at two centers (Campus Benjamin Franklin and Campus Virchow Klinikum, Charité University Medicine Berlin, Berlin, Germany) according to the following inclusion criteria: (i) aSAH of World Federation of Neurosurgical Societies (WFNS) grade I–V; (ii) age (≥ 18 years); (iii) ruptured saccular aneurysm proven by computed tomography (CT)-angiography or digital subtraction angiography; (iv) symptom onset within the preceding 72 h; (v) either surgical treatment of the aneurysm via craniotomy or, in coiled patients, burr hole trepanation for placement of a ventricular drain or oxygen sensor, which allows simultaneous placement of a subdural electrode strip (Bruce and Bizzi, 2000, Dreier et al., 2009, Eross et al., 2009). Exclusion criteria for patient monitoring were SAH due to other causes (e.g., trauma, fusiform or mycotic aneurysm), admission in a clinical state with unfavorable prognosis (e.g., wide, nonreactive pupils for > 1 h), bleeding diathesis or pregnancy, unavailability of the monitoring equipment and refusal of the patient or legal representative to participate in the study. The research protocol was approved by the local ethics committee of the Charité University Medicine Berlin. Either informed consent or surrogate informed consent was obtained for all patients. Research was conducted in accordance with the Declaration of Helsinki. The subdural electrode strip was targeted to the vascular territory of the aneurysm-carrying vessel because this is often covered with blood and, thus, a predilection site for delayed cerebral ischemia (Dreier et al., 2017).

Twelve of the 70 patients met further screening criteria for inclusion in the present study: (vi) at least one MRI without extensive pathology that would preclude successful cortical surface reconstruction using FreeSurfer (Martinos Center for Biomedical Imaging, Charlestown, MA, USA, http://surfer.nmr.mgh.harvard.edu/); (vii) at least one CT scan that displayed the collinear electrode strip; and (viii) the presence of SDs hitting at least 3 electrodes of the subdural strip, required for the algorithm. Demographic data for the 12 patients are shown in Table 1.

**Table 1:** Demographic data are given in the left part. ACA, anterior cerebral artery; ACoA, anterior communicating artery; MCA, middle cerebral artery; PCoA; posterior communicating artery. The patch section shows the properties of the geometric mesh for each patient. The variations in total patch area in the first column reflect the individual variations of surface folding at a constant distance (30 mm) between electrodes and any vertex on the edge of a given patch. The second column contains the cortical thickness in the patch. The quality of the geometric discretization before and after the up-sampling is reflected by the vertex count in the third and fourth column, and in the mean triangle edge length in the fifth and sixth column, respectively. The hemisphere section shows individual differences in total surface area and cortical volume of the individual brain hemispheres from which the single patches were extracted. The ECoG section gives the number of all recorded and analyzed SDs of the 12 patients and those SDs with at least three active electrodes, which was required for the algorithm.

No.	Age (years), sex	WFNS grade	Fisher grade	Location of aneurysm	Intervention	Focal brain lesion at electrode strip (either infarct or ICH)	Early focal brain lesion (days: 0–3)	Delayed focal brain lesion (days: 4–14)	Recording time [h]	Median SD velocity based on the reduced hit-sequences	Median interval to previous SD	PTDDD	Peak total number of SDs of a recording day	Peak total number of spreading depressions of a recording day	Peak total number of isoelectric SDs of a recording day	Patch						Hemisphere		ECoG	
																Area [cm^2^]	Thickness [mm]	Vertex count	Vertex count up-sampled	Mean edge length before up-sampling [mm]	Mean edge length after up-sampling [mm]	Area [cm^2^]	Volume [cm^3^]	Recorded SDs	Simulated SDs
1	56. f	4	3	MCA	Clipping	y	y	y	287.1	4,11	34.8	300.5	36.1	36.1	5.0	74.95	2.32 ± 0.69	10,526	165,713	0.96 ± 0.41	0.24 ± 0.10	1194.43	662.51	189	129
2	50, f	2	3	PCoA	Clipping	n	n	y	253.1	2,42	158.1	106.4	11.9	11.9	0.0	72.45	2.59 ± 0.62	9155	144,185	1.01 ± 0.43	0.25 ± 0.11	898.17	572.42	46	7
3	68, f	4	3	PCoA	Coiling	y	y	y	258.7	5,1	27.0	1103.8	47.1	13.2	36.1	82.10	2.16 ± 0.71	9800	154,208	1.03 ± 0.43	0.26 ± 0.11	1041.34	513.65	150	86
4	50, f	3	3	ACoA	Clipping	y	y	y	228.7	6,72	58.2	647.3	34.7	25.1	9.6	88.54	2.69 ± 0.85	10,185	159,765	1.05 ± 0.48	0.26 ± 0.12	1009.99	497.14	65	25
5	64, f	4	3	ACoA	Clipping	n	n	y	278.2	3,2	136.2	158.5	12.5	12.5	1.0	68.70	2.87 ± 0.94	8502	133,719	1.01 ± 0.47	0.25 ± 0.12	9899.50	569.43	58	45
6	58, f	1	3	ACoA	Clipping	n	y	n	287.3	3,84	19.8	389.6	37.1	24.7	12.4	70.80	2.76 ± 0.93	8623	135,421	1.03 ± 0.47	0.26 ± 0.12	9078.07	481.31	55	41
7	48, m	5	3	ACoA	Coiling	n	y	y	303.8	2,43	408.5	206.5	20.1	20.1	0.0	73.24	2.80 ± 0.71	9091	142,921	1.02 ± 0.46	0.26 ± 0.11	1151.69	429.90	77	5
8	31, m	2	3	ACoA	Clipping	y	n	y	202.1	2,6	61.6	410.0	20.9	20.9	5.1	79.79	2.47 ± 0.78	8720	136,796	1.09 ± 0.48	0.27 ± 0.12	1227.96	575.25	48	31
9	47, f	4	3	MCA	Clipping	y	y	y	269.3	3,22	103.8	502.4	21.4	13.2	21.4	90.72	2.33 ± 0.75	10,745	169,058	1.02 ± 0.45	0.26 ± 0.11	1154.76	502.34	99	72
10	44, f	4	3	PCoA	Clipping	y	y	y	239.9	3,28	31.8	1407.0	53.5	30.2	53.5	71.92	2.33 ± 0.77	8331	130,926	1.05 ± 0.46	0.26 ± 0.11	1041.35	397.84	174	124
11	70, f	4	3	ACA	Clipping	y	y	y	245.1	2,47	138.3	184.3	8.1	8.1	4.0	75.48	2.66 ± 0.74	9306	146,379	1.02 ± 0.48	0.26 ± 0.12	9576.65	456.25	27	25
12	61, f	1	3	PCoA	Clipping	n	y	n	256.1	3,18	267.2	179.0	12.1	12.1	7.1	72.46	2.59 ± 0.62	9155	144,185	1.01 ± 0.43	0.25 ± 0.11	8981.77	391.40	49	21
																								1037	611 (58.9%)

Aneurysmal SAH was diagnosed through interdisciplinary assessment of CT scans by a neuroradiologist and a neurosurgeon. Hemorrhage was graded according to the original Fisher scale (Fisher et al., 1980), and clinical presentation on admission according to the WFNS scale. A study neurologist or neurosurgeon performed a neurological and general medical evaluation on admission. Baseline demographic data and clinical signs and symptoms of the initial hemorrhage were recorded. The aneurysm was assessed using four-vessel digital subtraction angiography, or a more restricted study when indicated.

After aneurysm treatment by either clip ligation or endovascular coil embolization, all patients were transferred to the intensive care unit where the continuous neuromonitoring data were acquired for up to 15 days. Glasgow Coma Score, blood gases, glucose and electrolytes were documented at least every 6 h. A thorough neurological examination was performed at least daily. Oral nimodipine was given prophylactically. Transcranial Doppler-sonography was performed daily as described previously (Dreier et al., 2012). MRI exam protocol included a T2-weighted fluid-attenuated inversion recovery (FLAIR) sequence, a T1-weighted sequence pre- and post‑gadolinium, a T1-weighted 3D high resolution sequence (i.e. magnetization prepared rapid gradient echo (MPRAGE)), and a diffusion weighted imaging (DWI) sequence. The first MRI was performed 24-48 h after aneurysm treatment in order to assess the initial structural brain injury, the second MRI around day 7 and the third one on the day of electrode withdrawal (~ day 15) to assess the occurrence of delayed ischemic strokes during the ECoG monitoring period. In addition, serial CT scans were performed at times of clinical deterioration. At the conclusion of the monitoring period, the electrode strip was removed at the bedside by gentle traction.

### 2.2 ECoG acquisition

ECoG was recorded with a subdural collinear electrode strip carrying six disk-shaped platinum electrodes, each 4 mm in total diameter with 2 mm diameter of the contact surface and 10 mm distance between electrode centers (Ad-Tech Medical, Racine, Wisconsin, USA). The strip was placed on cortex after craniotomy (Fig. 1A) or through an extended burr-hole and connected to a GT-205 amplifier (0.01–100 Hz). The near-DC/AC-ECoG signal was sampled at 200 Hz with a PowerLab 16/SP analog/digital converter and LabChart software (all by ADInstruments, New South Wales, Australia). In parallel the electrode strip was connected to a DC-coupled BrainAmp amplifier (0–100 Hz) (Brain Products GmbH, Munich, Germany) and the recorded DC/AC-ECoG data imported into the LabChart software. All recorded signals were analyzed using monopolar montage against a subdermal platinum needle electrode placed over the hemisphere ipsilateral to the recording strip. DC/AC-ECoG data were used whenever possible. However, they were replaced by the near-DC/AC-ECoG data in case of recording problems, such as, for example, saturation of the DC amplifier due to drifting. Following current recommendations, SDs in electrically active tissue received the epithet “spreading depression”. By contrast, SDs measured in a zone of electrically inactive tissue were denoted with the adjective “isoelectric” (Fig. 1B) (Dreier et al., 2017).

### 2.3 Overview of the modeling approach

In order to reconstruct the potential SD trajectories and velocities based on the TOADs, we chose to restrict the trajectories to the ones for which the following assumptions held: (1) following the typically observed pattern in animal experiments (Dahlem and Müller, 2004, Kaufmann et al., 2017, Kneer et al., 2014, Santos et al., 2014, Scholl et al., 2017), the shape of the SD wave-front can be approximated by a circular arc that propagates tangentially along the discretized brain surface; (2) the radius of this arc is fairly constant; (3) the velocity of the arc's center is fairly constant; (4) the SD center travels on geodesics (shortest paths) from the point where it reaches one electrode to the point at which it reaches the next electrode in temporal succession.

The trajectory reconstruction was a model fitting procedure that searched in the class of models consistent with the assumptions as given above. These assumptions are simplifying. Therefore, there were real cases in which no model existed for the observed data and the trajectory reconstruction hence failed. In practically all successful searches the assumptions were not constraining enough to reduce the ambiguity to the point where a single trajectory fit the data; therefore, the reconstruction produced groups of many potential trajectories. In this context, we use “simulation” to describe the process of iterative extension (from electrode to electrode, in the order a given SD reached the electrodes) of the set of potential trajectories, because it corresponds to simulating/extending all those trajectories in parallel - not completing one trajectory to the last electrode hit before starting describing the key points of the next possible trajectory. The logical parallelism implemented by grouping the partial trajectories by their end point accelerates the search of the valid, observations-conform trajectories in the huge space of the geometrical trajectories. Assumption number (4) above is very strong, and is equivalent to requiring that the SD wave-front travelled at the lowest velocity to explain the observed TOADs. Without this assumption, any trajectory would be possible, although many of those would require high velocities that are not biologically plausible.

Between each two electrodes ***A*** and ***B*** (identified by their position on the cortex), hit in succession by the SD, all trajectories on the discretized cortical surface were (virtually) retained that were between the vertex (Fig. 1C) ***a*** in the neighborhood of ***A*** and the vertex ***b*** in the neighborhood of ***B*** (***a*** and ***b*** were candidate positions for the center of the SD), provided that the distance between ***a*** and ***A*** approximately matched the distance between ***b*** and ***B*** (their difference was upper-bounded by a search parameter, **R_diff**). This condition enforced the second assumption above. The velocity estimated for the trajectory segment ***a–b*** was given by the times the SD hit ***A*** and ***B*** and the geodesic distance on the cortical surface between ***a*** and ***b***, according to our fourth assumption.

Trajectory segments ***a–b*** (first electrode pair) and ***b–c*** (second electrode pair) were joined into an ***a–c*** trajectory if and only if their velocities approximately matched (maximum difference was upper-bounded by a search parameter, **V_diff**). This filtering enforced our third assumption. The process was repeated iteratively, extending the partial trajectories with every new electrode-to-electrode hop and retaining only those trajectories for which a suitable extension existed.

### 2.4 Imaging data preprocessing

Cortical reconstruction, including both gyri and sulci, was performed with FreeSurfer. In brief, this processing comprises removal of non-brain tissue (Segonne et al., 2004), automated Talairach transformation, segmentation of the subcortical white matter and deep grey matter volumetric structures (Fischl et al., 2002), intensity normalization, tessellation of the grey/white matter boundary, topology correction and surface deformation (Dale et al., 1999, Fischl and Dale, 2000) to produce representations of cortical thickness, which are calculated as the closest distance from the grey/white matter boundary to the grey matter/cerebrospinal fluid boundary at each vertex on the surface. The reconstructed discrete brain surface generated by FreeSurfer remains identical to the geometry of the resampled MRI images. The reconstructed cortical surface can then be converted into a geometric mesh by exporting it as an ASCII file with FreeSurfer's single-line command. The structure of such ASCII files is very similar to the off-format, which we used for the simulation after simple conversion with Matlab. As a result, the cortical surface is mathematically modeled by a grid of unequal triangles (= tessellation) (Fig. 1C). Such a surface is termed a discrete non-Euclidean or discrete two-manifold surface. The distance between two points (vertices) on a two-manifold surface is given as the shortest distance along the surface, called geodesic distance. We used Matlab (MathWorks Inc., Natick, MA, USA) for data processing and simulation, and the Dijkstra shortest path algorithm (contained in the BOOST Graphic Library (BGL) toolbox) for computing geodesic distances.

The electrodes from the CT images were obtained by simple thresholding since the brain tissue ranges between 20 and 120 Hounsfield Units (HU) and metallic parts have values over 3000 HU. The surface of isolated electrodes was geometrically reconstructed by Matlab's ‘isosurface’ function. To localize the electrodes on the geometric mesh representing the cortical surface, we first rigidly co-registered the CT scan with electrodes to the MRI scan reoriented and resampled with FreeSurfer. Secondly, we estimated the centroids of every reconstructed electrode using Matlab's ‘regionprops’ function. Finally, we identified the vertices on the cortical mesh with the closest Euclidean distance to the centroids. The final result was a discrete cortical surface in form of a triangulated mesh with the known electrode positions. This provided the spatial information for the simulation.

### 2.5 Geometric data preprocessing

We up-sampled the geometric mesh because of the rounding error that resulted from the inequality of triangles. To reduce the long computation time we ran the simulation using a patch (Fig. 1C). This patch was cut from the cortical mesh with 30 mm radius around each electrode. To ensure that the space between electrodes and any point on the patch's edge was never smaller than 30 mm, we computed the shortest paths between each neighboring electrode and checked the distances for every vertex on the path. Every patch was up-sampled by doubling the number of the vertices twice (Table 1) according to the spline interpolated 4-split method (Shirman, 1990). Up-sampling slightly increased the number of could-fit solutions and improved the results from the path search by Dijkstra's algorithm. The increase in number of vertices by up-sampling for factor K also increased the computation time required by a polynomial algorithm of order P for K^P times. The original mesh generated by FreeSurfer often contains small unnaturally edgy areas. We applied the Laplace operator to smooth the curvature and set the parameters in order to retain the original lengths of the edges and, thus, the original topology (Desbrun et al., 1999). Only the angles between edges were changed so that the smoothing predominantly affected the global curvature. From a vertex selected as a disc's center, we calculated shortest paths to every other vertex of the patch.

### 2.6 SD trajectory search procedure – parameters and details

The search for candidate trajectories of a given SD was performed in a stepwise fashion by considering sub-trajectories for each pair of neighboring electrodes that showed a time difference between SPC onsets indicating a time of arrival delay of the respective SD (= TOAD). The number of steps varied depending on the number of TOADs which ranged between two and five, or, respectively, a minimum of three and a maximum of six active electrodes. A variable wave-front between sub-trajectory sets was possible within the limits given by the maximally allowed wave-front curvature radius difference (R_diff as introduced above) and wave-front velocity difference (V_diff as introduced above), respectively. Since the path search presumed a constant curvature radius and velocity of the wave-front within a single sub-trajectory, R_diff and V_diff allowed the simulation to compensate for small changes in velocity, wave-front curvature and spatial shear between two neighboring sub-trajectory sets and a given electrode. A stepwise search for the sets of sub-trajectories between individual electrode pairs also allowed the simulation to compensate for more complex shapes of trajectories than the simple ones based exclusively on the shortest path search.

Pre-set maxima of R_diff and V_diff were used to filter the connecting candidates belonging to two consecutive sub-trajectory sets. Candidates exceeding either of these two parameters were excluded. Given a sufficiently large maximum radius, the region adjacent to each electrode was considered with all vertices within the preset disk radius. The bipartite distances between each pair of vertices were pre-computed and the trajectories were then iteratively “grown” starting from zero-length partial trajectories and proceeding stepwise according to the order of incidence. The zero-length trajectory seeds were all the vertices in the region of the first electrode hit by the SD (step 1). At step N all vertices around the corresponding electrode were considered as possible disk centers separately for each velocity subrange. All possible pairs between currently possible partial trajectory endings around the (N − 1)th hit electrode and the vertices in the region of the Nth hit electrode were considered with exclusion of pairs at which (i) the segment velocity did not fit the fixed velocity subrange, or (ii) the distance to the (N − 1)th electrode differed too greatly from the distance to the Nth electrode.

The accepted velocity range was set between 0 and 15 mm/min per sub-trajectory set with a tolerance range V_diff between the sets. V_diff was set in turn at three different values (0.25, 0.5, and 1 mm/min). The accepted wave-front curvature radius was set between 0 and 5 mm per single set with a tolerance range R_diff between the sets. R_diff was set at two different values (0.5 and 1 mm). Finer granulation of V_diff values was not necessary since the number of solutions dropped dramatically at lower tolerance values. The wave-front radii of the simulation tended to be very conservative in contrast to the velocity between the sub-trajectory sets. The reason to set a lower limit for R_diff at 0.5 mm was the average edge length at around 0.25 mm as shown in Table 1. The upper limits for both V_diff (in mm/min) and R_diff (in mm) were set at value 1. We repeatedly simulated for every pair of control parameters V_diff and R_diff, respectively. These numerical choices were a trade-off between computation time and the method's sensitivity. Furthermore, the selected upper limit was a trade-off between realistic and unrealistic trajectory candidates based on our knowledge from animal experiments. Every trajectory found was possible under the modeling assumptions and consistent with the observed TOADs between electrode pairs.

The velocity of every SD was then calculated as a weighted arithmetic mean of the estimated velocities over the electrode-to-electrode segments, with the TOADs as weights. The SD velocity over an electrode-to-electrode segment was calculated as the arithmetic mean of the sub-trajectories' velocities on that segment, after quantization toward the central values of the velocity sub-ranges. The velocity sub-ranges were defined for V_diff = 1 mm/min as ± 1 mm/min intervals around fifteen central values from 1 to 15 mm/min in 1 mm/min steps, resulting in 2 mm/min intervals with 50% overlap between every two neighbor ones.

### 2.7 Validation of results

In order to validate the explanatory accuracy of the simulation model, we withheld part of the available information by removing one of the electrode hits by a given SD and estimated the electrode hit times on a random selection of 100 trajectories from the ones proposed, taking the predicted SD radius and the predicted SD propagation velocity into account. Some of the trajectories generated for this reduced set of electrodes could no longer make a hit on the withheld electrode. The first measure of quality of the modeling was the percentage of cases where the predicted trajectory still predicted the withheld electrode being reached by the SD area during its propagation. The second measure of quality concerned the precision with which the electrode hit time matched the observed time for the withheld electrode corrected for velocity. This can be given as a spatial error between the trajectory from the actual simulation and the validation trajectory.

### 2.8 Statistics

Data are given as median (1st, 3rd quartile). In the figures, the whiskers (error bars) above and below the box indicate the 90th and 10th percentiles. Statistical tests are given in the text. *P* ≤ 0.05 was accepted as statistically significant. Note that only measurements averaged over patients can be considered independent. To get statistically valid results, two strategies were applied: (1) Characteristics of SDs of the same patient were summarized by medians calculated patientwise. Thus, significances are not inflated by possible cluster effects of measurements within the same patient. (2) In some cases, pooled analyses with “n” equal to the number of SDs were performed in addition when this provided additional information about the structure of the data as explained in the discussion. In these analyses linear mixed models were then applied with patient as random factor. To approach normal distribution, values were logarithmically transformed for these analyses related to SDs.

Standard ECoG analyses (Dreier et al., 2017) were performed by MW, blinded to the clinical courses, neuroimaging findings and simulation results. VK assessed the serial neuroimages for type of lesion, location with respect to electrode strip and time point of infarct or hemorrhage occurrence, blinded to the clinical courses, ECoG analyses and simulation results. DM determined the TOADs and analyzed the DC data under supervision of JD, and developed the algorithm in collaboration with CG and MD. DM's analysis of the DC shifts was based on the recently published criteria by Hartings and colleagues (Hartings et al., 2017a). DM, MW and VK were blinded to each other. JD and PM performed the statistical analysis.

## 3 Results

**Fig. 2:**
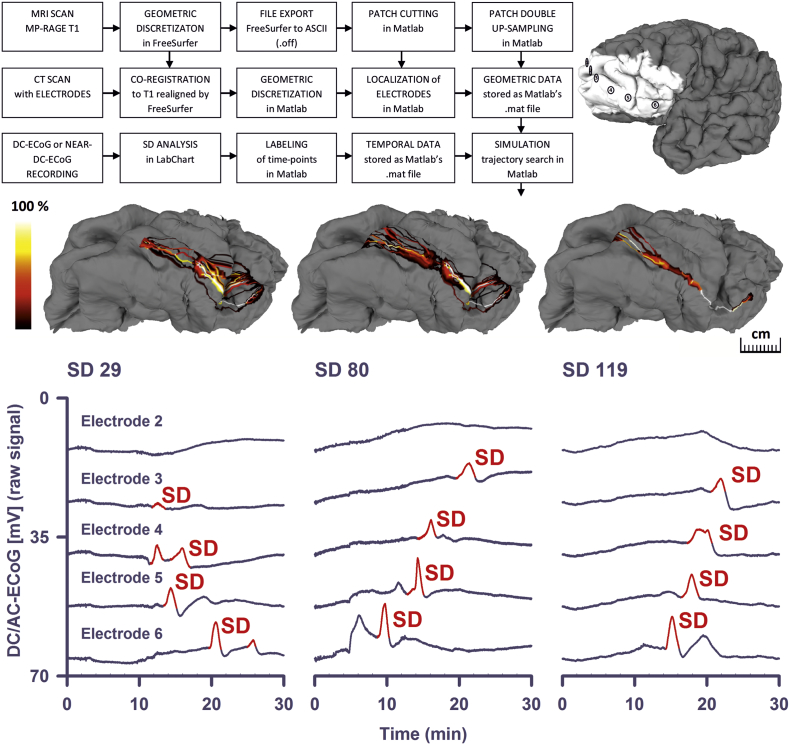
Simulations of SD trajectories using full-hit sequences. In the upper left corner, the processing pipeline up to the trajectory search is shown. In the upper right corner, the reconstructed surface of patient 1's brain is given including the subdural electrodes 1–6 and the patch around the electrodes. The next row demonstrates the trajectory search for three example SDs of patient 1 in the patch. Overlapping ‘could fit’ trajectories are color-coded as a heatmap in % relative to the total number of ‘could fit’ trajectories. This means that the higher the number of ‘could fit’ solutions including a given vertex on the brain surface for an electrode pair, the lighter the color of the respective vertex in the heatmap. In the lowest row, the original DC recordings of the three example SDs are given.

In 12 patients, our simulations found potential trajectories (‘could fit’ solutions) for 374 of 611 SDs (61.2%) using V_diff = 1 mm/min and R_diff = 1 mm. Using lower tolerance values the number of successful simulations decreased (Table 2). The median count of ‘could fit’ solutions per SD was 4.18 × 10^16^ (1st quartile, 3rd quartile: 1.43 × 10^12^, 3.58 × 10^18^ trajectories). Fig. 2 shows the results of the trajectory search for three SDs in patient 1. In these examples, overlapping ‘could fit’ trajectories are color-coded as a heatmap.

**Table 2:** This table shows the number of successfully reconstructed SD events per subject and per combination of simulation parameters. R_diff represents the maximally allowed absolute difference for the curvature radius of a wave-front between any candidate in the preceding and any candidate in the succeeding sub-trajectory set. V_diff represents the maximum absolute velocity difference in otherwise the same way as R_diff does. A sub-trajectory set is related to the surface between a pair of consecutively activated electrodes in which the trajectory reconstruction is possible according to the propagation model. The upper half of the table labeled as ‘full hit-sequence’ contains SD reconstruction counts based upon the time-lags, so called TOADs, directly adopted from the ECoG recordings by the labeling of SD events. The lower half labeled as ‘reduced hit-sequence’ contains results based upon the hit-sequences with removed branches.

Subject	Simulated SDs	R_diff = 1 V_diff = 1	R_diff = 1 V_diff = 0.5	R_diff = 1 V_diff = 0.25	R_diff = 0.5 V_diff = 1	R_diff = 0.5 V_diff = 0.5	R_diff = 0.5 V_diff = 0.25
Full hit-sequences							
1	129	66 (51.2%)	54 (41.9%)	42 (32.6%)	66 (51.2%)	53 (41.1%)	36 (27.9%)
2	7	2 (28.6%)	2 (28.6%)	2 (28.6%)	2 (28.6%)	2 (28.6%)	2 (28.6%)
3	86	64 (74.4%)	31 (36.0%)	26 (30.2%)	64 (74.4%)	30 (34.9%)	26 (30.2%)
4	25	23 (92.0%)	20 (80.0%)	20 (80.0%)	22 (88.0%)	20 (80.0%)	20 (80.0%)
5	45	25 (55.6%)	24 (53.3%)	24 (53.3%)	26 (57.8%)	24 (53.3%)	23 (51.1%)
6	41	32 (78.0%)	30 (73.2%)	30 (73.2%)	32 (78.0%)	30 (73.2%)	29 (70.7%)
7	5	4 (80.0%)	3 (60.0%)	2 (40.0%)	4 (80.0%)	2 40.0%)	2 (40.0%)
8	31	30 (96.8%)	29 (93.5%)	26 (83.9%)	30 (96.8%)	28 (90.3%)	26 (83.9%)
9	72	27 (37.5%)	25 (34.7%)	20 (27.8%)	27 (37.5%)	25 (34.7%)	20 (27.8%)
10	124	67 (54.0%)	65 (52.4%)	61 (49.2%)	67 (54.0%)	64 (51.6%)	60 (48.4%)
11	25	15 (60.0%)	13 (52.0%)	12 (48.0%)	15 (60.0%)	13 (52.0%)	12 (48.0%)
12	21	19 (90.5%)	18 (85.7%)	18 (85.7%)	19 (90.5%)	18 (85.7%)	18 (85.7%)
All	611	374 (61.2%)	314 (51.4%)	283 (46.3%)	374 (61.2%)	309 (50.6%)	274 (44.8%)

Reduced hit-sequences							
1	129	112 (86.8%)	105 (81.4%)	94 (72.9%)	112 (86.8%)	104 (80.6%)	88 (68.2%)
2	7	4 (57.1%)	4 (57.1%)	3 (42.9%)	4 (57.1%)	4 (57.1%)	3 (42.9%)
3	86	65 (75.6%)	32 (37.2%)	26 (30.2%)	65 (75.6%)	31 (36.0%)	26 (30.2%)
4	25	23 (92.0%)	21 (84.0%)	21 (84.0%)	22 (88.0%)	21 (84.0%)	21 (84.0%)
5	45	40 (88.9%)	39 (86.7%)	39 (86.7%)	41 (91.1%)	39 (86.7%)	38 (84.4%)
6	41	27 (65.9%)	26 (63.4%)	26 (63.4%)	27 (65.9%)	26 (63.4%)	25 (61.0%)
7	5	4 (80.0%)	3 (60.0%)	2 (40%)	4 (80.0%)	2 (40.0%)	2 (40.0%)
8	31	31 (100.0%)	30 (96.8%)	27 (87.1%)	31 (100.0%)	29 (93.5%)	27 (87.1%)
9	72	52 (72.2%)	50 (69.4%)	45 (62.5%)	53 (73.6%)	50 (69.4%)	45 (62.5%)
10	124	98 (79.0%)	92 (74.2%)	87 (70.2%)	97 (78.2%)	92 (74.2%)	85 (68.5%)
11	25	25 (100.0%)	24 (96%)	24 (96.0%)	25 (100.0%)	24 (96.0%)	23 (92.0%)
12	21	21 (100.0%)	21 (100%)	21 (100.0%)	21 (100.0%)	21 (100.0%)	21 (100.0%)
All	611	502 (82.2%)	447 (73.2%)	415 (67.9%)	502 (82.2%)	443 (72.5%)	404 (66.1%)

**Fig. 3:**
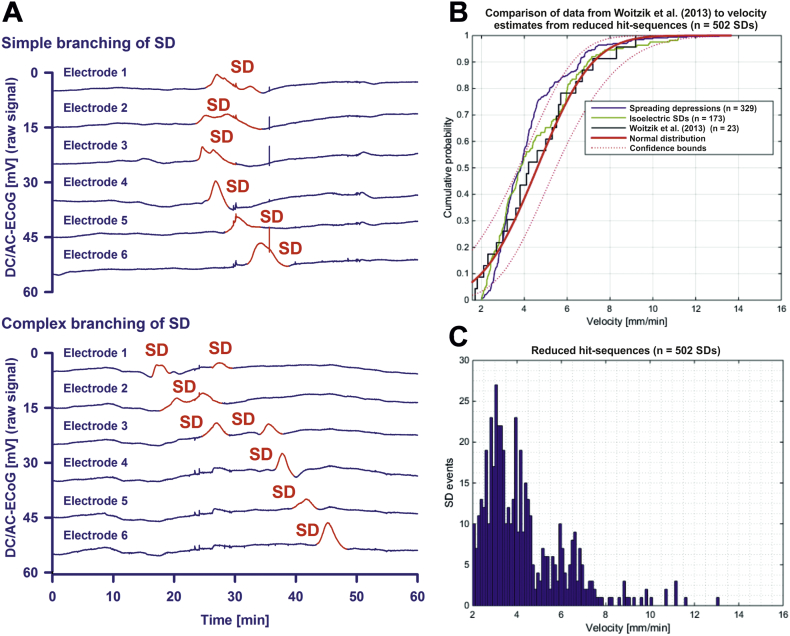
Simulation-estimated velocity based on the reduced hit-sequences. (A) Simple branching of SDs was frequently observed. For example in the upper SD, the full hit-sequence E2-E3-E4-E1-E5-E6 is a twin sequence starting at E2. From E2, one of the wave's branches hits E3-E4-E5-E6 and the other one E1. In cases of branching, we removed either the shorter branch or the one that occurred later when the two branches were of the same length. In this case, E1 was removed. For this reduced hit-sequence, the trajectory search was successful. The lower SD from the same patient shows a more complex type of branching. In this case, no trajectories were found for either full or reduced hit-sequences. This example SD is particularly interesting because the unique moment was recorded at which it subdivided and appeared in form of a double peak DC shift in E2. Such double peak DC shifts could be longer than 4 min, though they might not indicate local energy compromise. In the context of branching, we would like to refer previous videos of SDs in the gyrencephalic brain of swine in which this is visualized using IOS imaging (Santos et al., 2014, Scholl et al., 2017). Neuroimaging excluded in this case that E1, E2 or E3 sampled from two adjacent gyri. (B) Using laser speckle imaging of rCBF and IOS imaging in the operating room, Woitzik and colleagues recorded SD velocities between 1.7 and 9.2 mm/min in patients undergoing decompressive hemicraniectomy (Woitzik et al., 2013). These historical data were fitted here to normal distribution and are compared with the simulation-estimated velocities of spreading depressions and isoelectric SDs based on the reduced hit-sequences. (C) Frequency distributions of the simulation-estimated velocities for the reduced hit-sequences.

For the 374 SDs, the median of the trajectory velocity was 3.6 (2.9, 5.2) mm/min. Since the simulation found ‘could fit’ trajectories for only 61.2% of the total number of SDs, we also ran the simulation with simplified SDs whenever branching or even more complex, non-longitudinal hit-sequences occurred (Fig. 3A). Branching describes a type of SD in which the depolarization first arrived on any of the electrodes in the middle of the strip (electrode 2 (E2), E3, E4 or E5) and continued from there in two different directions. In cases of branching, we removed either the shorter branch or the one that occurred later when the two branches were of the same length. Under this condition, the number of SDs in which the simulation found ‘could fit’ solutions rose from 374 to 502 of 611 (82.2%) for V_diff = 1 mm/min and R_diff = 1 mm (Table 2). For these reduced hit-sequences, the median count of ‘could fit’ solutions per SD was 1.43 × 10^12^ (1.01 × 10^11^, 3.60 × 10^15^ trajectories). The median velocity was similar to the one based on the full hit-sequences (3.6 (2.8, 4.8) mm/min). The velocity histograms in Fig. 3C represent the velocities of all successfully simulated SDs from all subjects at the tolerance values R_diff = 1 mm and V_diff = 1 mm/min for the reduced hit-sequences.

The validation procedure was then performed for all subjects and all successfully simulated SDs using one hundred randomly selected trajectories per electrode triad within a single SD. The number of validation steps for a single SD depended on the total number of active electrodes. Validation results were solely related to the middle electrodes from the electrode triads. The results from the single steps of the validation procedure were aggregated with equal weights. As the first measure of quality we calculated the fraction of successful validations relative to the total number of validation attempts. This was 90.4% (338 out of 374 SDs) for the full hit-sequences and 94.8% (476 out of 502 SDs) for the reduced ones. The second measure of quality was the spatial error between the validation trajectories and the trajectories from either the full or reduced hit-sequence simulations. The spatial error was − 2.2 (− 0.1, − 4.2, n = 338) mm with respect to the full hit-sequences and − 1.9 (0.2, − 4.1, n = 476) mm with respect to the reduced ones.

**Fig. 4:**
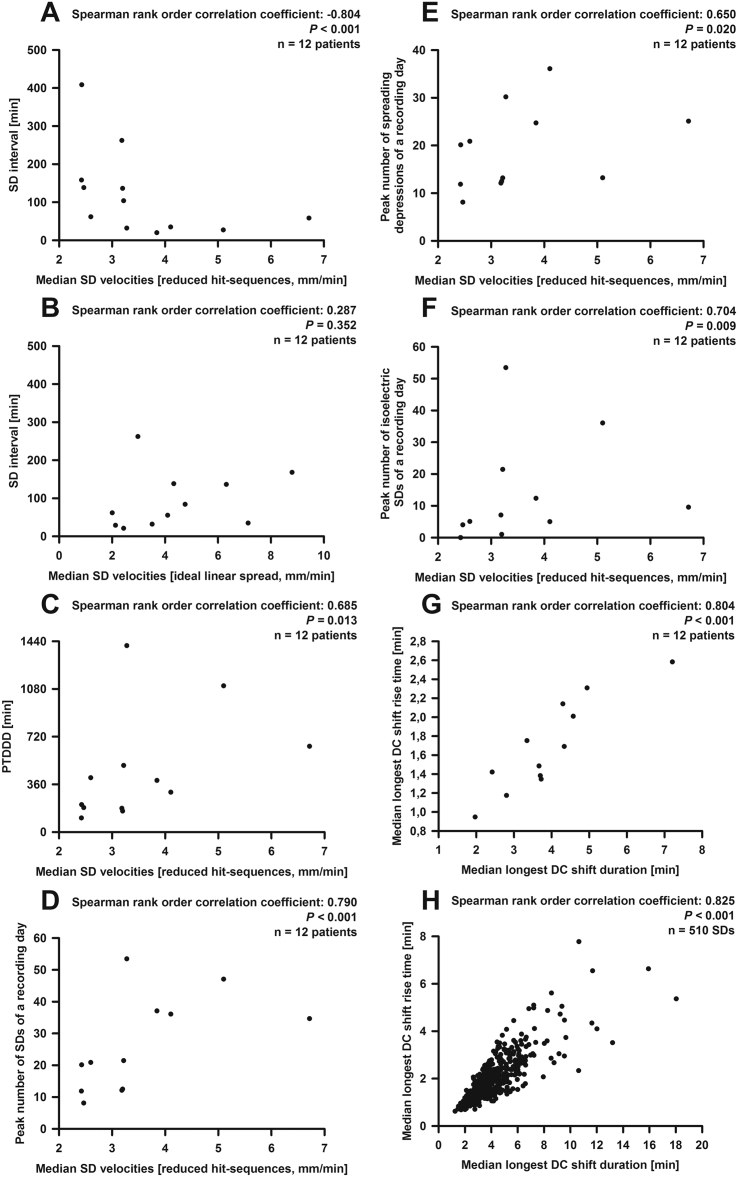
Statistical analyses of the simulation-estimated velocity based on the reduced hit-sequences. (A) A significant correlation of median velocity (based on the reduced hit-sequences) and median interval between SD and previous SD was found. (B) By contrast, there was no correlation of median interval between SD and previous SD with the median velocity based on an ideal linear spread along the recording strip (using the inter-electrode space of 10 mm). This is noteworthy because this type of velocity has been used in all previous COSBID publications that reported SD velocities to indicate the spread of the wave. Significant correlations of the median velocity (based on the reduced hit-sequences) were also found with (C) the PTDDD, (D) the peak number of SDs, (E) the peak number of spreading depressions and (F) the peak number of isoelectric SDs. (G) DC duration and DC rise time showed a strongly positive correlation when the medians were taken. (H) However, the correlation was even stronger using the pooled data which corresponds to the notion that the nature of this relationship is less complex than the relationship between SD velocity and susceptibility.

The third and most important measure of quality was the biological validation using variables that were not involved in the simulation procedure. For this purpose, we investigated whether the median estimated velocities based on the full and reduced hit-sequences correlated with typical variables related to the tissue's susceptibility to SD. Notably a high negative correlation was found between the median SD velocity based on the reduced hit-sequences and the median interval between SD and the previous SD (Fig. 4A). Significant correlations were also found with the peak total SD-induced depression duration of a recording day (PTDDD) (Dreier et al., 2017) (Fig. 4C) and the peak numbers of SDs (Fig. 4D), spreading depressions (Fig. 4E) and isoelectric SDs (Fig. 4F) of a recording day. No significant correlation was found with age. The median SD velocities based on the full hit-sequences correlated significantly with the median interval between SD and the previous SD (Spearman rank order correlation coefficient: − 0.58, *P* < 0.045, n = 12 patients) and the peak number of SDs of a recording day (Spearman rank order correlation coefficient: 0.62, *P* = 0.031, n = 12 patients) but not with the other variables (number of correlations = 3). The median wave-front velocities assuming an ideal linear spread along the recording strip, using either the inter-electrode space of 10 mm or the shortest path along the brain surface, did not correlate with any of these variables (Fig. 4B).

We also investigated whether SD velocities based on the reduced hit-sequences and the SD interval between SD and the previous SD correlated when all successfully simulated SDs were pooled. Though this correlation was statistically significant, it was relatively weak (Spearman rank order correlation coefficient: − 0.10, *P* = 0.035, n = 497 SDs). Moreover, in the double logarithmic linear mixed model, taking into account single SDs and correcting for cluster effects, influences of speed and SD interval were not significant (*P* = 0.113).

The median longest DC shift among all electrodes of a given SD was 3.7 (3.2, 4.4) min, the median longest DC rise time was 1.6 (1.4, 2.0) min and the median largest DC amplitude 5.9 (4.1, 7.2) mV (n = 12 patients). DC duration and DC rise time showed a strongly positive correlation (Fig. 4G). This was also highly significant for the pooled data (Fig. 4H); the linear mixed model revealed a highly significant effect (*P* < 10^− 100^). Only the pooled data showed significant correlations of the DC shift amplitude with the DC shift duration (Spearman rank order correlation coefficient: 0.20, linear mixed model: *P* < 0.0001, n = 509 SDs). The correlation between DC shift amplitude and DC rise time was not significant in the linear mixed model (*P* = 0.092, n = 509 SDs).

Velocities of isoelectric SDs and spreading depressions were not significantly different (3.9 (3.0, 4.8, n = 9 patients) versus 3.1 (2.6, 3.8, n = 12 patients) mm/min, reduced hit-sequences). Also their velocity distributions were similar. They showed a good fit with previous measurements of SD velocities in patients with MHS undergoing decompressive hemicraniectomy Fig. 3B) (Woitzik et al., 2013). Isoelectric SDs demonstrated a significantly lower SD-SD interval than spreading depressions in electrically active cortex (29.6 (26.6, 53.7, n = 10 patients) min versus 113.7 (44.3, 178.7, n = 12 patients) min, *P* = 0.011, Mann-Whitney Rank Sum Test) similar to previous observations in patients with aSAH (Winkler et al., 2017). Moreover, they showed a significantly higher number of DC shifts lasting longer than 4 min (124 out of 174 isoelectric SDs (71.3%) versus 80 out of 336 spreading depressions (23.8%), *P* < 0.001, Chi-Square Test) similar to previous observations in patients with aSAH and TBI (Hartings et al., 2011b, Oliveira-Ferreira et al., 2010).

**Fig. 5:**
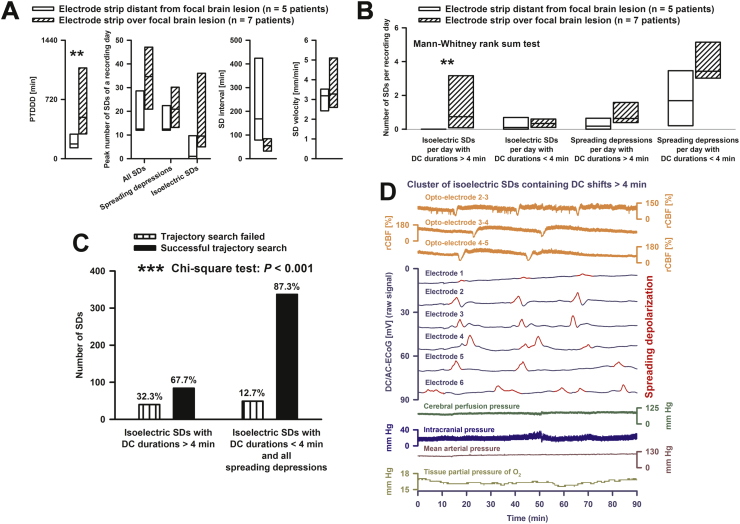
Comparison between recording areas undergoing structural damage (n = 7 patient) and recording areas distant from zones undergoing structural damage (n = 5 patients). (A) Among the standard variables recommended by the COSBID group (Dreier et al., 2017), a significant difference was found only for PTDDD. However, the statistical power of these tests was low. (B) Isoelectric SDs with DC shifts > 4 min were only observed in patients in whom the electrode strip was overlying either a primary or secondary focal brain lesion. This corresponds well with the animal literature (Hartings et al., 2017b). (C) The simulation based on the reduced hit-sequences failed to find possible SD trajectories in a significantly higher proportion of isoelectric SDs with DC shift durations > 4 min compared with the remaining SDs. (D) The third SD of three consecutive SDs in a cluster of isoelectric SDs with DC shift durations > 4 min is an example, taken from patient 10, that illustrates the statistical results in (C). The simulation failed to find possible SD trajectories in this SD, no matter whether full or reduced hit-sequences were used. The first three traces show short-lasting spreading ischemias in response to the SDs (Dreier et al., 2009). Regional CBF was measured with a subdural opto-electrode strip that allowed the simultaneous measurement of ECoG and rCBF using laser-Doppler flowmetry (Perimed AB, Järfälla, Sweden) (Dreier et al., 2009, Drenckhahn et al., 2016). Cerebral perfusion pressure results from the subtraction of the intracranial pressure (monitored via ventricular drainage catheter) from the mean arterial pressure (catheter in the radial artery). Tissue partial pressure of oxygen was recorded using an intraparenchymal sensor (Licox CC1P1, Integra Lifesciences Corporation, Plainsboro, NJ, USA) (Bosche et al., 2010, Dreier et al., 2009, Hinzman et al., 2014, Winkler et al., 2017). ** indicates that the *P*-value or probability value for the statistical comparisons given in the figure is < 0.01 when the null hypothesis is true; *** indicates that it is < 0.001 when the null hypothesis is true (cf. body text for the applied statistical tests).

**Fig. 6:**
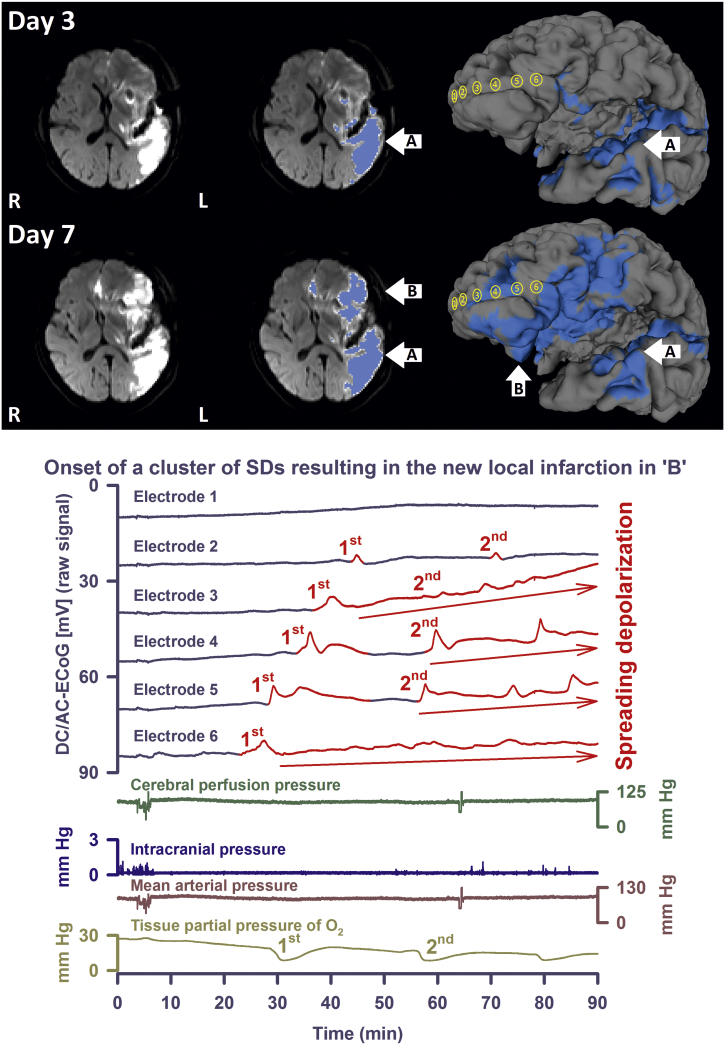
Development of a delayed ischemic infarct after aSAH in patient 3. Upper: Diffusion-weighted MRI (DWI) shows an infarct in the posterior territory of the left middle cerebral artery (MCA) on day 3 (upper left image). On day 7, a new delayed ischemic infarct is visualized in the left anterior MCA territory including the recording area (lower left image). In the middle images, the ischemic lesions are marked in blue (A = early and B = delayed infarct). The blue regions of interest originate from DWI (b = 1000) images superimposed onto geometrically discretized (triangular mesh) whole brain taken as MPRAGE sequence from the same subject. The region of interest threshold was set at 2/3 of the maximum value. On the right side, the reconstructed brain surfaces are depicted. Also on the reconstructed cortical surfaces, the DWI lesions are marked in blue. The subdural recording strip was projected from a CT onto the cortical surface (yellow electrodes 1 to 6). Note that, apart from electrode 1, all electrodes overlay the new delayed infarct. Lower: DC/AC-ECoG recordings of the two initial SDs of a cluster that occurred on day 4 after aSAH between the two MRIs of days 3 and 7. Based on our knowledge of the electrophysiological signature of stroke in animal experiments, the cluster was presumably the correlate of the new infarct. Whereas the simulation found possible trajectories for the first SD, it failed to do so for the second one. This provides another example illustrating that the simulation did not find possible SD trajectories in a significantly higher proportion of isoelectric SDs with DC shift durations > 4 min compared with the remaining SDs. Also note that the SDs are superimposed on a negative ultraslow potential (red arrows) as explained recently (Dreier et al., 2017). Traces are similar to Fig. 5.

Isoelectric SDs with DC shifts > 4 min were only observed in patients in whom the electrode strip was overlying either a primary or secondary focal brain lesion (n = 7) but not in patients in whom the electrode strip was located remote from focal brain lesions (n = 5) (*P* = 0.010, Mann-Whitney Rank Sum Test, Fig. 5B, Table 1). The simulation based on the reduced hit-sequences failed to find possible SD trajectories in a significantly higher proportion of these isoelectric SDs with DC shift durations > 4 min compared with the remaining SDs (*P* < 0.001, Chi-Square Test, Fig. 5C). This suggests a higher proportion of SDs with more complex propagation patterns in this subgroup. Figs. 5D and 6 illustrate these results using example SDs measured in the recording area during the evolution of delayed ischemic infarcts. The median velocity of isoelectric SDs with DC shifts > 4 min was 3.4 (2.5, 3.9, n = 6 patients) mm/min and not different to that of the remaining SDs.

In addition to the occurrence of isoelectric SDs with DC shift durations > 4 min, recording areas with primary or secondary focal brain lesions (n = 7 patients) also differed significantly from recording areas remote from focal brain lesions (n = 5 patients) in the PTDDD (502.4 (355.2, 875.6) versus 179.0 (158.5, 206.5), *P* < 0.018, Mann-Whitney Rank Sum Test), whereas differences in the other variables, as shown in Fig. 5A, did not reach statistical significance.

## 4 Discussion

Collinear subdural electrode strips are used in neurocritical care rather than electrode grids though grids would ease the calculation of SD velocities. This is because strips can be removed at the bedside by gentle traction whereas withdrawal of grids requires another neurosurgical intervention in the operating room with the known potential risks for the patient. Yet in order to estimate SD velocities in neurocritical care, an algorithm was developed here. For the biological validation of this, we exploited the well-established relationship between SD velocity and the tissue's susceptibility to SD based on animal experiments. For example, the hippocampal area CA3 of immature rabbits is less susceptible to SD than area CA1, which was attributed to a higher Na,K-ATPase activity of area CA3 (Haglund et al., 1985). The Na,K-ATPase blocker ouabain was then applied at a concentration in which it completely inhibits the α_2_/α_3_ isoforms and partially inhibits the α_1_ isoform (Major et al., 2017). This not only evened out the difference in SD susceptibility between the two areas but also facilitated the invasion of SD from area CA1 to area CA3 (Haglund and Schwartzkroin, 1990). A significantly increased SD velocity in parallel with an increased susceptibility to SD was found in genetically modified mice, in which astrocyte-directed inactivation of Cx43 decreased astrocytic gap junctional communication (Theis et al., 2003). A similar correlation was also found in mouse models of familial hemiplegic migraine type 1, a rare Mendelian model disease of migraine with aura (Cain et al., 2017, van den Maagdenberg et al., 2004). Further, this link was noted in acquired metabolic disruptions. For example, pathologically low concentrations of nitric oxide (NO) enhanced the susceptibility to SD in vivo and in brain slices in rodents (Petzold et al., 2008, Petzold et al., 2005), and conversely, elevated NO reduced the propagation velocity of SD in the chicken retina (Ulmer et al., 1995). Because of the experimentally and clinically well-established decline in NO availability following aSAH, the latter may have particular relevance to the patient population investigated in this study (Dreier, 2011, Pluta et al., 2009). Also various drugs including anesthetics with concurrent effects on SD velocity and susceptibility might have relevance to our patient population (Dhir et al., 2012, Kudo et al., 2008, Marrannes et al., 1988). The same applies to female hormones and their modulatory effects because the majority of aSAH patients are women (Eikermann-Haerter et al., 2009). Finally, SD velocity and susceptibility showed concurrent changes with age. Thus, young rats were more prone to develop SDs and SD velocities were higher (Hablitz and Heinemann, 1989, Maslarova et al., 2011, Menyhart et al., 2015). In the present study, the influence of age was however not statistically significant. This may have several reasons: only adults were included, the study was relatively small and other modulatory factors may be stronger in this severely diseased patient population.

The propagation process of SD is discussed controversially as reviewed recently (Dreier and Reiffurth, 2015). Accordingly, also the question of how SD velocity and susceptibility are mechanistically linked has not been fully elucidated. However, the propagation process is generally regarded as a reaction/diffusion rather than a simple diffusion mechanism (Zandt et al., 2013). It is thus assumed that neurons release neuroactive substances such as potassium or glutamate, which diffuse to adjacent neurons where they trigger a self-propagating regenerative process. This concept entails the re-induction of SD at every spot in the tissue reached by the SD wave and might therefore well explain why SD velocity and susceptibility correlate.

### 4.1 SD propagation shows anisotropy

The propagation of SD usually shows deviations from concentricity in both liss- and gyrencephalic brains (Kaufmann et al., 2017, Santos et al., 2017). An apparent tropism of SD for superficial layers, rather than deeper layers of cortex, has been repeatedly reported (Basarsky et al., 1998, Bogdanov et al., 2011, Grafstein, 1956, Herreras and Somjen, 1993, Richter and Lehmenkuhler, 1993). Cytoarchitectonic peculiarities also affect the propagation in the horizontal plane (Chen et al., 2006, Eiselt et al., 2004, Leão and Morison, 1945). The exact mechanisms underlying this anisotropy have remained enigmatic but myelin content, neuron-astrocyte ratio and vascular anatomy have all been advanced as possible explanations (Fujita et al., 2016, Merkler et al., 2009, Santos et al., 2014). The present observations in humans are in line with this anisotropy in animals. In particular, we found a relatively high proportion of SDs that branch or propagate heterogeneously, as found previously with imaging of the gyrencephalic swine brain (Santos et al., 2014). The frequency of branching necessitated use of the algorithm with the reduced rather than the full SD hit-sequences.

Intervals between repeated SD waves also vary with propagation patterns and susceptibility. For instance, the first SD in the otherwise healthy gyrencephalic brain of cats usually spreads with an elliptical wavefront over the ipsilateral cerebral hemisphere whereas succeeding SDs often remained within the originating gyrus, were slower, more fragmented and varied in number (James et al., 1999). A similar decline in susceptibility to SD after the first event was also observed in rodents (Chen et al., 2006, Kaufmann et al., 2017, Sukhotinsky et al., 2011). Accordingly, imaging studies in migraineurs suggested that an isolated SD often affects large parts of the hemisphere (Hadjikhani et al., 2001, Lauritzen, 1994, Olesen et al., 1981, Woods et al., 1994) although the representation fields responsible for the visual percept seem to be relatively small (Dahlem and Hadjikhani, 2009). However, repeated SDs in humans were often restricted to a single gyrus as assessed with laser speckle imaging in the operating room in patients with MHS (Woitzik et al., 2013).

Nonetheless, velocity and interval to the previous SD correlated only weakly in our study when every SD was considered in form of a pooled SD-related (versus patient-related) analysis. A strong correlation was merely found between these variables in the patient-related analysis based on medians, thus averaging the data. This result supports strong constitutional (e.g. genetic, disease- or drug-related) components with general influence on both variables rather than a direct dependence between these two variables. In this context it is worth noting that the recorded interval to the preceding SD depends above all on the local conditions where a given SD arises (Dreier et al., 2017, Winkler et al., 2017), but depends only secondarily on the transmission of the SD from its site of origin to the recording site. By contrast, the recorded velocity is merely determined by the conditions at the recording site.

In contrast to the correlation of velocity and interval to the previous SD, not only medians of DC durations and DC rise times but also pooled data showed highly significant correlation. This suggests a direct dependence of these two variables, which are merely determined by the local tissue conditions at the recording site. This statistical result is consistent with observations that the ultraslow negative potential of prolonged SDs does not usually ascend abruptly in subdural recordings (Dreier et al., 2017).

### 4.2 More complex propagation patterns of isoelectric SDs with prolonged DC shifts

It has been established in numerous animal studies that the local duration of the DC shift is energy-dependent, indicates the local duration of the cytotoxic edema and, hence, the risk of injury at the recording site (Back et al., 1994, Dijkhuizen et al., 1999, Hartings et al., 2003, Nallet et al., 1999, Oliveira-Ferreira et al., 2010). Moreover, it has been noted for decades that zones with prolonged negative DC shifts become electrically inactive (Astrup et al., 1981, Hossmann, 1994, Koroleva and Bures, 1996, Oliveira-Ferreira et al., 2010). For the first time, we here provide corresponding statistical evidence in patients, based on MRI findings and ECoG recordings, that isoelectric SDs with prolonged DC shifts exclusively occur in recording areas undergoing irreversible damage. The velocity of such SDs was not significantly different from the velocity of other SDs. Largely overlapping SD velocities between well-nourished and energetically compromised tissue correspond well with previous in vitro and in vivo studies in rodents (Aitken et al., 1998, Bere et al., 2014a, Farkas et al., 2010, Jarvis et al., 2001). These findings render it unlikely that changes in SD velocity are useful as an early warning sign of impending ischemia in the individual patient. However, trajectory reconstruction of those SDs failed significantly more often, according to the notion based on animal experiments that their propagation paths can become exceedingly complex (Bere et al., 2014b, Nakamura et al., 2010, Scholl et al., 2017). In concert with other parameters such as the SD frequency and the durations of depressions and DC shifts (Dreier et al., 2017), this could possibly be used as a tool to monitor alarming development in the individual patient. The strongest deviation from concentricity is their reverberation due to continuous cycling. This was first demonstrated in rats in vivo by Shibata and Bures who performed a surgical lesion of the cortex to impose a pathway of cyclical propagation (Shibata and Bures, 1972). A similar approach also resulted in cycling in the isolated chicken retina (Martins-Ferreira et al., 1974). Cycling of SD was moreover observed around electrically stimulated areas, epileptic foci or ischemic zones (Koroleva and Bures, 1979, Nakamura et al., 2010). It has been advanced as an explanation for the periodicity in clusters of SDs (Nakamura et al., 2010, Santos et al., 2017, Scholl et al., 2017).

### 4.3 Limitations

A limitation of our study was the small size with 12 patients. This was due to the fact that the MRI segmentation sequence of FreeSurfer is optimized for analyzing human brains free of pathology. However, an exceedingly large number of the 70 screened patients had significant pathologies including grey matter edema or missing parts of the skull. This often rendered FreeSurfer unable to geometrically reconstruct the cortical surface. Even manual correction of the segmentation frequently failed. Moreover, the simulation required at least three active electrodes but patients often showed either no SDs or SDs hitting less than three recording electrodes.

We should also like to indicate that we only used a simple arc as wave front on the discretized brain surface although the front of SD typically shows a depolarized zone of time-varying width both in liss- and gyrencephalic animal cortex whose spatial extent locally depends (i) on the time necessary for the energy-dependent repolarization after the local SD onset, (ii) on the local velocity, and (ii) probably also on the curvature of the local gyrification pattern (Dahlem and Müller, 2004, Kaufmann et al., 2017, Kneer et al., 2014, Santos et al., 2014, Scholl et al., 2017). It is, however, unlikely that the widths of the depolarized zones significantly impact on the velocities because, in animals, SDs under normoxic and hypoxic conditions strongly differ in widths of the depolarized zones but SD velocities are similar (Aitken et al., 1998). Yet the shape of the wave fronts might still deviate more from a simple arc in zones undergoing injury. This could be among the reasons why the simulation more often failed to find possible SD trajectories for isoelectric SDs with DC shifts > 4 min than for other SDs.

The class of trajectories that we considered was limited according to the four assumptions we made. Whereas the first three assumptions have a biological justification, assumption (4) that the trajectory can abandon a geodesic (“change direction”) only when the SD hits an electrode is rather artificial and is a compromise between the limited data available for model fitting and the richness of the models.

Another limitation was that the onset of SD at a given electrode can be subjective. However, all simulations were completed before the statistical analysis started. Further, simulation and statistical analysis were performed by different investigators so that the statistical analysis could not influence the TOADs on which the simulations were based.

## 5 Conclusions

We established a novel algorithm enabling the estimation of SD velocities in patients monitored in neurocritical care. The validity of the algorithm was supported by the fact that the fundamental relationship between SD velocity and susceptibility was similar to that reported in experimental animals. Our findings establish the opportunity to exploit this variable as part of the multimodal assessment in neurocritical care. For example, it was found in previous studies that the peak number of SDs of a recording day was significantly higher in patients who later developed post-hemorrhagic epilepsy (Dreier et al., 2012) and that isoelectric SDs and a high PTDDD were associated with poor patient outcome (Dreier et al., 2012, Hartings et al., 2011a, Winkler et al., 2017). Based on the correlations identified here, it would be interesting to investigate whether a high median SD velocity is also associated with these risks. However, in order to render this variable available to a larger patient population, better methods for geometrical reconstruction of brains with severe pathology would be required.
